# Enhanced activity of the left precuneus as a predictor of visuospatial dysfunction correlates with disease activity in rheumatoid arthritis

**DOI:** 10.1186/s40001-023-01224-1

**Published:** 2023-08-09

**Authors:** Yanmin Zheng, Lei Xie, Zikai Huang, Jianhua Peng, Shuxin Huang, Ruiwei Guo, Jinzhuang Huang, Zhirong Lin, Zelin Zhuang, Jingjing Yin, Zhiduo Hou, Shuhua Ma

**Affiliations:** 1 Department of Radiology, The First Affiliated Hospital of Shantou University Medical College, No. 57 ChangPing Road, Shantou, 515041 Guangdong China; 2 Department of Rheumatology, The First Affiliated Hospital of Shantou University Medical College, Shantou, China; 3 Laboratory of Medical Molecular Imaging, The First Affiliated Hospital of Shantou University Medical College, Shantou, China

**Keywords:** Visuospatial dysfunction, Rheumatoid arthritis, Disease activity, Precuneus, Functional MRI

## Abstract

**Objective:**

To identify the potential diagnostic biomarkers of rheumatoid arthritis (RA) and assess the relation between visuospatial dysfunction and disease activity in RA patients using mental rotation task (MRT)-based functional magnetic resonance imaging (fMRI).

**Methods:**

A total of 27 RA patients (11 in remission, 16 in active) and 27 well-matched controls were enrolled. The visuospatial function of the subjects was measured by MRT. Brain activity data were collected using blood oxygen level dependent fMRI technique under MRT. Disease activity score 28 (DAS28) was used to evaluate the disease severity of RA patients. An analysis of the correlations between abnormal visuospatial-related brain regions, MRT performance, and DAS28 was conducted.

**Results:**

RA patients performed worse on MRT than controls. Compared to the control group, RA patients showed enhanced activation in the left precuneus, left superior frontal gyrus and right cingulate gyrus during the rotation task, with left hemisphere dominance. RA patients in active showed enhanced activation in the left precuneus, left middle frontal gyrus and right cingulate gyrus compared to the patients in remission. The left precuneus activation was negatively correlated with MRT accuracy (*r* = −0.621, *p* = 0.01) and positively correlated with DAS28 (*r* = 0.710, *p* = 0.002), and MRT accuracy was negatively correlated with DAS28 in RA patients (*r* = −0.702, *p* = 0.002).

**Conclusion:**

Enhanced activation of the left precuneus in RA patients affects visuospatial function and is closely related to disease activity. These changes may provide a valuable diagnostic neuroimaging biomarker of RA.

## Introduction

Rheumatoid arthritis (RA) is a chronic multi-system inflammatory disease, and its typical clinical symptoms are symmetric multiple arthritis, mainly involving small joints [1]. The global prevalence of RA is approximately 0.5–1.0%, and is more common in women [2]. Inflammatory and immune responses involve all systems in RA patients [3]. The disease activity score in 28 joints (DAS28) is commonly used to assess disease activity in RA. This score is based on the number of swollen joints, the number of tender joints, erythrocyte sedimentation rate (ESR) and C-reactive protein (CRP), and the overall patient evaluation by a visual analog scale [4]. The primary goal of RA treatment is to achieve remission and low disease activity. DAS28 plays an important role in disease assessment, medication guidance and prognosis. Other systems are also damaged in RA patients, leading to various complications, including damage to the nervous system and cognition dysfunction.

Bartolini et al. found that visuospatial tasks were impaired and highly correlated with disease activity in 71% of RA patients [5]. Shin et al. found 31% of RA patients are cognitively impaired [6]. A 20-year cohort study of RA patients showed worse cognitive status in their middle age [7]. Visuospatial function can serve as a screening factor for cognitive disorders such as Alzheimer disease and Huntington disease [8, 9]. Some researchers believe that infiltration of inflammatory cells in RA patients leads to the increased load of small vessels and affects the supply of blood oxygen to the central nervous system, thus causing cognitive dysfunction [3]. At present, most studies on cognitive impairment of RA adopt neuropsychological scales, but there is a lack of neuroimaging markers for the diagnosis of visuospatial cognitive impairment in RA. Therefore, the incidence and pathogenesis of visuospatial dysfunction in RA remain unclear. BOLD-fMRI (blood oxygen level dependent-functional magnetic resonance imaging), widely used in neuroscience research, plays an indispensable role in neurocognitive science due to its non-invasive, high spatiotemporal resolution and high sensitivity [10]. Task-based fMRI detects neural activity based on comparison between activation conditions to control conditions by using a blocked design or event-related design [11]. The mental rotation task (MRT) is a well-known task for assessing visuospatial function, which involves the ability to mentally transform three-dimensional objects in space [12].

We hypothesized that there existed brain functional changes associated with disease activity and that could serve as neuroimaging biomarker of cognitive impairment in RA patients. Herein we used mental rotation task-based fMRI combined with psychological scales to investigate visuospatial cognitive function in RA patients and further explore whether the changes in brain functional characteristics are associated with disease activity of RA.

## Participants and methods

### Participants

In this cross-sectional study, 29 female patients with RA were recruited from the Department of Rheumatology, the First Affiliated Hospital of Shantou University Medical College, during the period of December 2019 to April 2021. Among the RA patients, 2 were excluded due to excessive head motion. Inclusion criteria for the RA group were as follows: meeting the 2010 American College of Rheumatology and European Alliance Against Rheumatology (ACR/EULAR) classification criteria for rheumatoid arthritis [13], ages between 18 and 60, education ≥ 6 years, right-handed, normal vision or corrected-to-normal vision, able to cooperate in cognitive function tests. Exclusion criteria were if the patient had other types of rheumatic immune system diseases, organic brain disorders, other systemic chronic diseases, mental illness or family history of chronic mental illness and cognitive impairment, drug and alcohol dependence, MRI contraindications and head motion more than 2 mm or 2 during MR scanning. All RA patients in this study were enrolled by two rheumatologists according to the above criteria. Healthy control subjects (HCs) comprised 27 female volunteers who matched the RA patients in age, sex, handedness and education level.

This study was approved by the Ethics Committee of the First Affiliated Hospital of Shantou University Medical College (B-2021-237). All participants gave written informed consent.

### Neuropsychological and clinical assessments

Cognitive functions were assessed by the Montreal Cognitive Assessment (MoCA), which includes visuospatial/executive, naming, attention, language, abstraction, delayed recall and orientation subtests. People are considered to have normal cognitive function if the score is ≥ 26, mild cognitive impairment if < 26, and dementia if ≤ 19 [14]. The Hospital Anxiety and Depression Scale (HAD) is a 14-item questionnaire used to assess levels of anxiety (7 items) and depression (7 items). Anxiety and depression are scored separately. Higher scores indicate a more severe mood disorder: normal = 0–7, mild = 8–10, moderate = 11–15, and severe = 16–21 [15]. The Health Assessment Questionnaire (HAQ) is used to evaluate the physical state of RA patients [16]. The Disease Activity Score (DAS) combines information from swollen joints, tender joints, acute phase response and patient self-report of general health to measure the disease activity of RA patients [17]. The DAS28 is a modified and reliable assessment, which is widely used in clinical practice. A DAS28 < 2.6 means patients are in remission, while a DAS28 ≥ 2.6 means patients are in active [4]. Both DAS28-CRP and DAS28-ESR were used in this study. The neuropsychological scales were evaluated by two psychologists, and the HAQ and DAS28 were completed by two rheumatologists.

### Mental rotation task and experimental procedure

The block-designed MRT was programmed and presented using E-Prime 3.0 software (Psychology Software Tools Inc; PST, Pittsburgh, PA, USA). The 3D graphics, designed by Shepard and Metzler [11], were presented to participants during the fMRI scan. There were two types of tasks: comparison and rotation. In the rotation block, a picture had two 3D drawings that were not on the same plane and were placed at a certain rotation angle (100°). Such two images were called identical graphics if they could be completely overlapped after rotation transformation, while those that could not be completely overlapped were different images. In the comparison task, two drawings were in the same plane with no rotation angle. If the two drawings were identical, participants were required to press the left button, otherwise the right button was pressed.

**Fig. 1:**
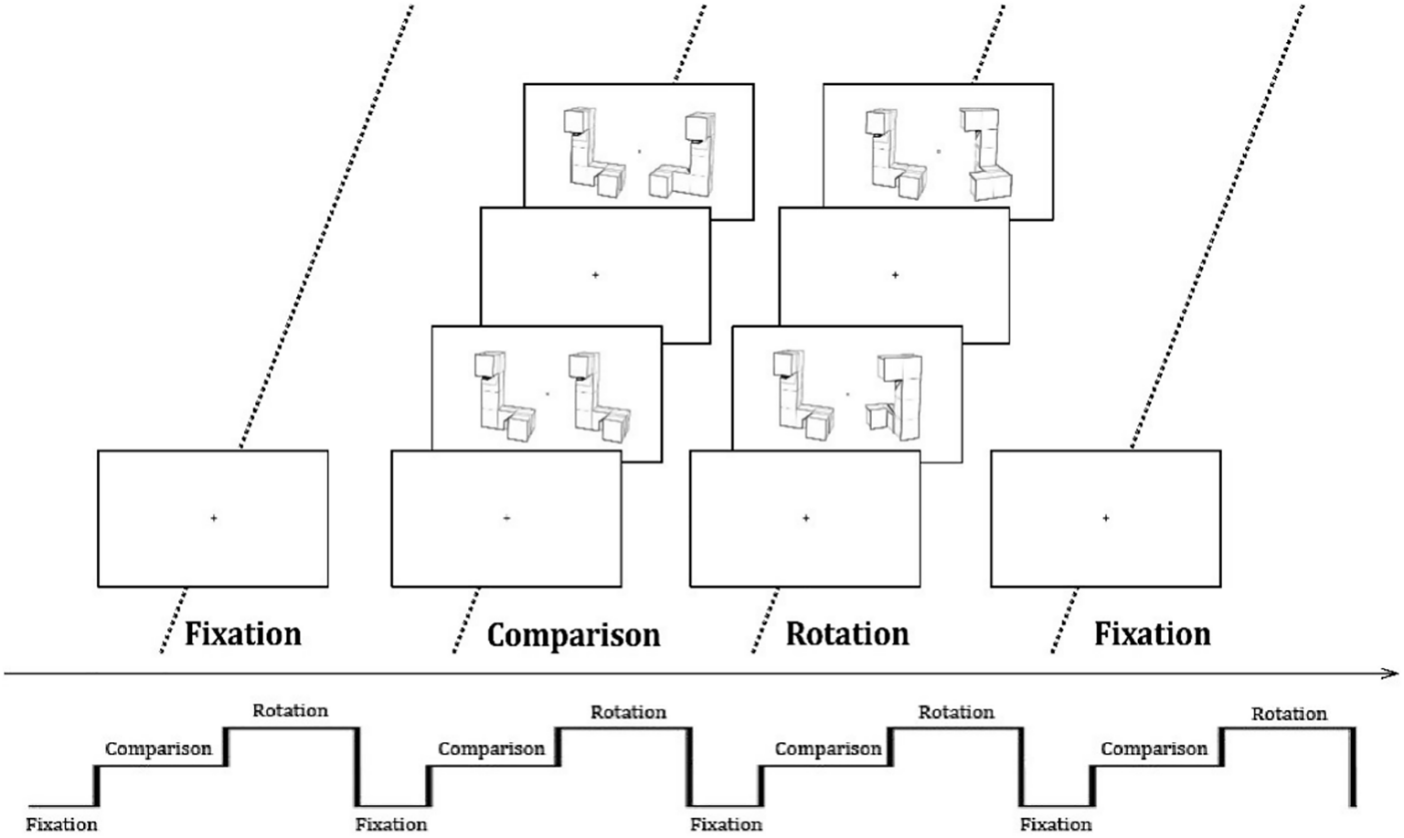
Block design of the mental rotation task. Fixation, comparison and rotation blocks were alternately presented 4 times during the entire experiment. A cross was displayed in the center of the screen for 30 s in each fixation block. Each block of the comparison or rotation blocks included 12 trials, lasting 84 s. Each trial consisted of a baseline condition (a screen with a center fixation presented for 0.5 s) and a stimulus (presented for 6.5 s)

There were four epochs in an fMRI scan. Each epoch consisted of a fixation block (a screen with a fixation cross lasting 30 s), a comparison block and a rotation block. Each task block included 12 trials, each of which contained a baseline condition (a screen with a center fixation presented for 0.5 s), and a task-related stimulus (presented for 6.5 s). So, the total experimental duration was 13 min and 12 s. The experimental procedure is shown in Fig. 1.

All participants were given instructions and training session before the fMRI scan. The E-Prime and stimulus presentation goggles system (SA-9939, Shenzhen Sinorad Medical Electronics Co., Ltd.) were used to present the stimulus. MRT was performed synchronously during the MRI scan, and the accuracy and response time were recorded by E-prime.

### fMRI image and data acquisition

Imaging data were acquired using a 1.5 T MR scanner (GE Medical Systems, Milwaukee, WI, USA) and a standard GE head coil in the Department of Radiology, the First Affiliated Hospital of Shantou University Medical College. Headphones and earplugs were provided to shield background noise. BOLD-fMRI acquisition used an echo-planar imaging sequence with the following parameters: repetition time (TR) = 3000 ms, echo time (TE) = 45 ms, flip angle (FA) = 90°, imaging matrix = 64 × 64, 20 slices, field of view (FOV) = 250 mm × 200 mm, slice thickness = 6.0 mm, no gap. Slices were acquired in an interleaved method in order to avoid crosstalk between adjacent slices. Anatomical T1-weighted fast spoiled gradient recalled (FSPGR) image parameters: TR = 1.6 ms, TE = 5.1 ms, FA = 20°, matrix = 256 × 256, FOV = 256 × 256 mm^2^, 244 slices, slice thickness = 1.3 mm, and no gap.

### Image processing

We analyzed fMRI data using the Analysis of Functional NeuroImages (AFNI), which can preprocess, analyze, and display fMRI data (http://afni.nimh.nih.gov/afni/). The first 2 time-points of the fMRI images were removed. Preprocessing steps included slice timing correction, head motion correction, spatial smoothing (FWHM = 6 mm) and spatial normalization to standard coordinates of the Talairach and Tournoux atlas. Participants who had obvious head movements (motion > 2 mm or rotation > 2°) during scanning were excluded. A general linear model (GLM), with covariates of six motion, was used to model task-related effects in first-level individual analysis. We treated the fixation conditions as baseline and deconvolved two task-related regressors with the canonical hemodynamic response function (HRF).

A 2 × 2 factorial design analysis of variance (ANOVA) was designed for fMRI analyses, with the group (RA and HC group) and task (rotation and comparison) as factors. The differences of activation were analyzed for the main effect and simple effect of the group, task and the interaction effects of group by task. Statistical threshold was set as *p* < 0.05 (false discovery rates, FDR-corrected). A cluster size correction (20 voxels) was applied calculated from the 3dClustSim of AFNI on data initial threshold, voxel size of the analysis space was 3 × 3 × 3 mm^3^.

### Statistical analysis

The independent-samples t test was used to compare age, duration of education, and performance of neuropsychological tests between two groups. For accuracy and response time in the mental rotation task, a 2 × 2 factorial design ANOVA was used to investigate the main effect and interaction effect; an independent-samples t test was used between groups, and a paired t test was used within a group. Pearson correlation analysis was used to investigate the relationship among the ROIs of the activated brain region in a rotation task (left precuneus, left superior frontal gyrus and right cingulate gyrus), clinical and MRT data. Differences were considered statistically significant if *p* < 0.05 (two tailed). Continuous variables were expressed as mean ± standard deviation. Statistical analysis was performed with Statistical Package for the Social Sciences (SPSS) version26 for Windows (IBM, Armonk, NY, USA).

## Results

### General information

Demographic, clinical and neuropsychological data of all participants are shown in Table 1. There was no difference in age and education level between RA patients and HCs. The average MoCA score of RA patients was lower than that of the HCs (*p* < 0.001). The scores of visuospatial/executive, naming, attention, language, abstraction and delayed recall in the RA group were lower than those of HCs (all *P* < 0.01). Both scores of anxiety and depression in the RA group were higher than that of HCs (all *P* < 0.01), while RA patients did not meet the positive criteria of clinical anxiety/depression.

**Table 1:** Demographic, clinical and neuropsychological data

	RA group (*n* = 27)	HC group (*n* = 27)	*P*-value
Demographic and clinical characteristics			
Age (years)	44.81 ± 9.30	40.81 ± 9.00	0.114
Education (years)	8.93 ± 3.57	9.63 ± 3.45	0.465
Gender (female/male)	27/0	27/0	–
Years of disease	8.67 ± 5.62	–	–
ESR (mm/h)	42.81 ± 31.24	–	–
CRP (mg/L)	19.56 ± 18.19	–	–
HB (g/L)	109.89 ± 16.81	–	–
RBC (×10^12^/L)	4.18 ± 0.63	–	–
WBC (×10^12^/L)	8.22 ± 2.10	–	–
PLT (×10^9^/L)	333.67 ± 77.37	–	–
DAS28-CRP	3.01 ± 1.37	–	–
DAS28-ESR	3.49 ± 1.38	–	–
HAQ	0.16 ± 0.27	–	–
Neuropsychological test scale scores			
Visuospatial/executive function	3.41 ± 0.93	4.78 ± 0.42	< 0.001**
Naming	2.70 ± 0.54	3.00 ± 0.00	0.009**
Attention	5.19 ± 1.11	5.89 ± 0.32	0.004**
Language	1.78 ± 0.42	2.37 ± 0.49	< 0.001**
Abstraction	1.78 ± 0.42	2.00 ± 0.00	0.011
Delayed recall	3.41 ± 1.19	4.93 ± 0.27	< 0.001**
Orientation	5.85 ± 0.60	6.00 ± 0.00	0.21
MoCA scores	24.96 ± 3.14	29.48 ± 0.85	< 0.001**
HAD-A anxiety score	4.44 ± 3.33	1.52 ± 1.37	< 0.001**
HAD-D depression score	3.96 ± 2.31	1.51 ± 1.34	< 0.001**

Continuous variables are expressed as mean ± standard deviation

*ESR* erythrocyte sedimentation rate, *HAQ* Health Assessment Scale, *CRP* C-reactive protein, *Hb* hemoglobin, *RBC* red blood cells, *WBC* white blood cells, *PLT* platelets, *MOCA* Montreal Cognitive Assessment Scale, *HAD-A* Hospital Anxiety and Depression Scale-Anxiety Subscale, *HAD-D* Hospital Anxiety and Depression Scale-Depression Subscale

^**^p < 0.01

### Behavioral performance in the mental rotation task

MRT accuracy and response time of RA and HC groups are summarized in Table 2. There was no significant in the interaction effect of group by task (*F*_(1,104)_ = 0.243, *p* = 0.623) for response time, while the main effect of task (*F*_(1,104)_ = 66.012, *p* < 0.001) and main effect of group (*F*_(1,104)_ = 8.234, *p* < 0.001) were observed, with the rotation response time longer than the comparison response time. The interaction effect of group by task for accuracy (*F*_(1,104)_ = 33.58, *p* < 0.001) was detected, as well as the main effect of group (*F*_(1,104)_ = 194.29, *p* < 0.001) and task (*F*_(1,104)_ = 49.388, *p* < 0.001) for accuracy. Furthermore, the accuracy of RA patients was lower than that of HCs both in the comparison task (*t* = −5.517, *p* < 0.001) and the rotation task (*t* = −14.624, *p* < 0.001). As for RA patients, the rotation accuracy was lower than comparison accuracy (*t* = −7.12, *p* < 0.001), but no difference was found in the control group (*t* = −1.90, *p* = 0.66 > 0.05).

**Table 2:** Accuracy and reaction time of the mental rotation task in the RA and HC groups

	RA group (*n* = 27)		HC group (*n* = 27)	
	Comparison	Rotation	Comparison	Rotation
Response time (ms)	2319.62 ± 743.01	3428.98 ± 912.40	1896.64 ± 612.76	3112.44 ± 665.84
Accuracy (%)	84.56 ± 10.88	62.24 ± 10.83	95.88 ± 4.22	93.85 ± 4.69

### fMRI results

**Fig. 2:**
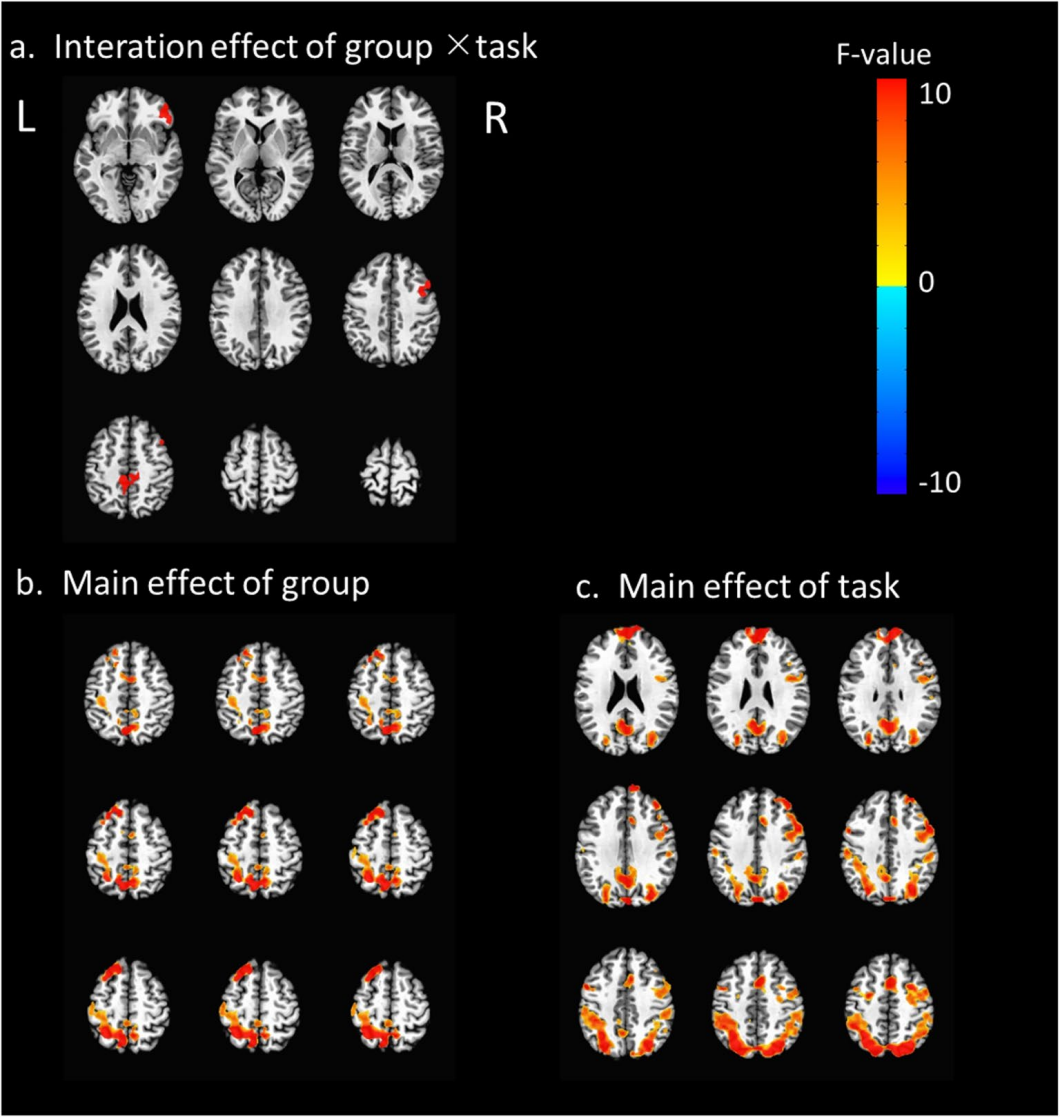
The interaction effect of group by task and the main effect of task/group. **a** Interaction effects: there were significant interaction effects, of each group by task, between the right inferior parietal gyrus, bilateral precuneus and right middle frontal gyrus; **b** main group effect of the RA group versus control group: the RA group showed significant clusters of activation in the precuneus, right superior frontal gyrus, and left cingulate gyrus; **c** main effect of task with rotation versus comparison: MRT produced significant clusters of activation in the precuneus, bilateral medial frontal gyrus, right anterior cingulate, left posterior cingulate and right precentral gyrus. *R* right hemisphere; *L* left hemisphere

**Fig. 3:**
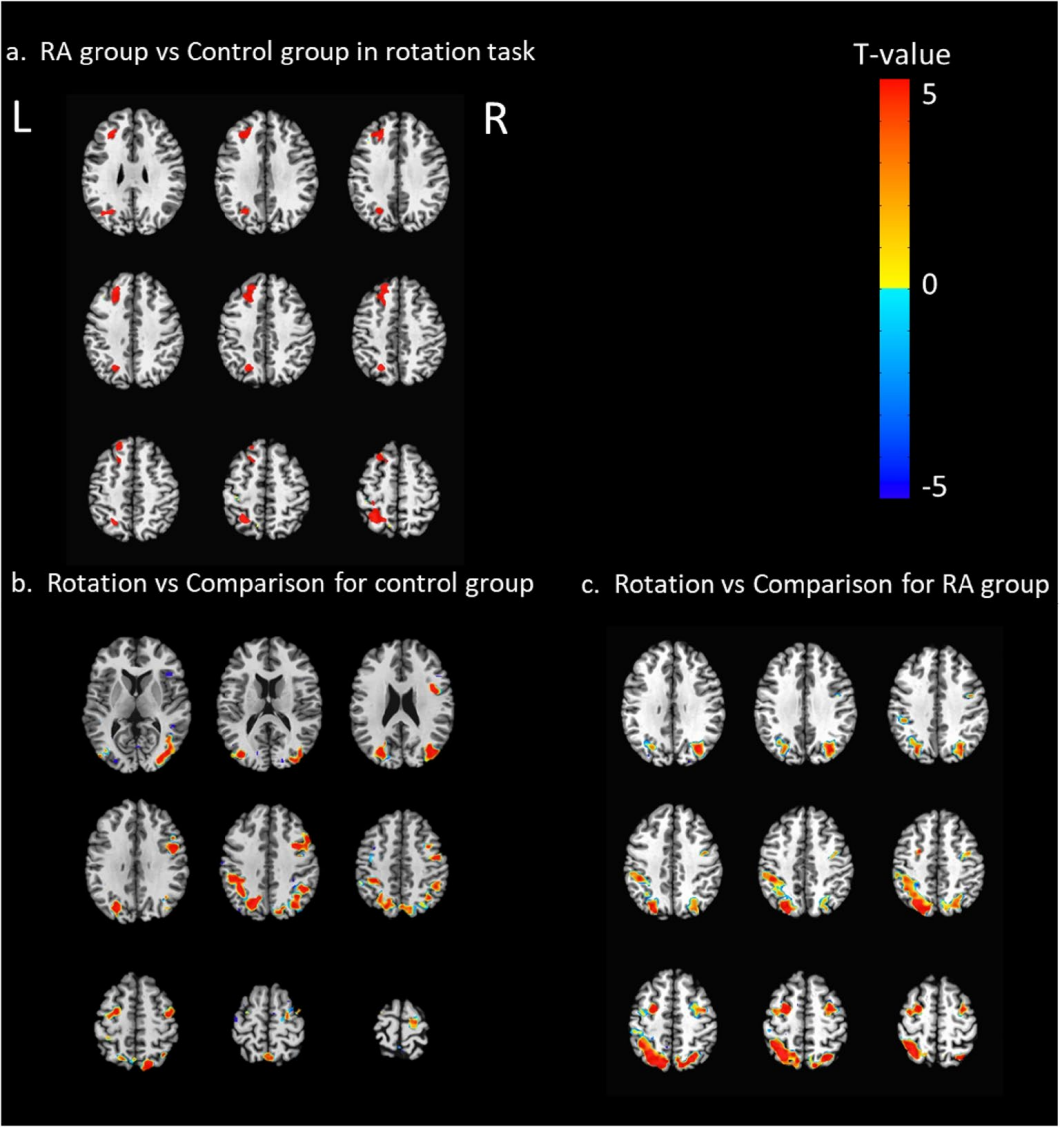
Brain activation differences activated in rotation test. **a** The RA group showed significantly increased activity in the left precuneus, left superior frontal gyrus and cingulate gyrus compared to the controls; **b** for the control group, active brain regions were involved the bilateral middle occipital gyrus, bilateral precuneus, right middle frontal gyrus and right precentral gyrus;** c**. for the RA group, the bilateral precuneus, left inferior occipital gyrus and bilateral middle frontal gyrus were detected and the left hemisphere was more active

**Fig. 4:**
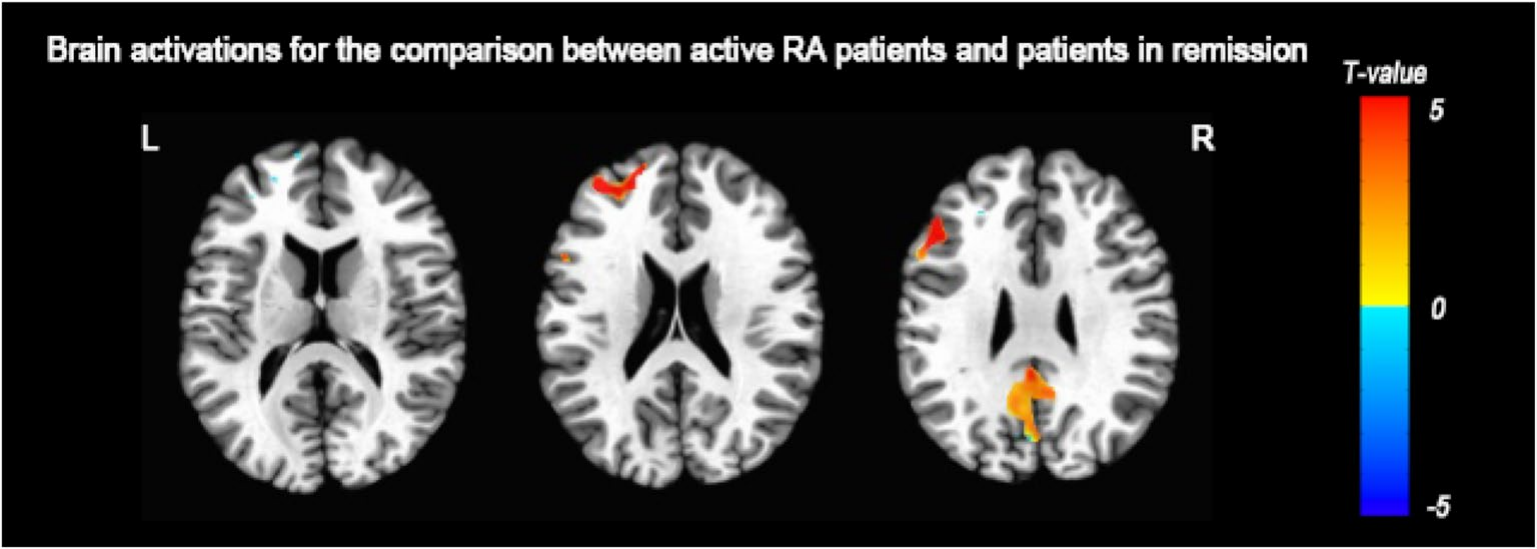
Brain activations for the comparison between RA patients in active and in remission. RA patients in active (DAS28 ≥ 2.6) showed enhanced activation the left precuneus, left middle frontal gyrus and right cingulate gyrus compared to the patients in remission (DAS28 < 2.6)

There was significant interaction effect of group by task in the right inferior parietal gyrus, bilateral precuneus and right middle frontal gyrus (Fig. 2a). For the main effect of group, enhanced activations were found in the precuneus, right superior frontal gyrus, left cingulate gyrus (Fig. 2b). For the main effect of task, significant clusters of activation were shown in the precuneus, bilateral medial frontal gyrus, right anterior cingulate, left posterior cingulate and right precentral gyrus (Fig. 2c, Table 3). For control group, there were activations for rotation effect in the bilateral middle occipital gyrus, bilateral precuneus, right middle frontal gyrus and right precentral gyrus (Fig. 3b). For the RA group, bilateral precuneus, left inferior occipital gyrus and bilateral middle frontal gyrus were active and the left hemisphere was more active (Fig. 3c). Compared to the control condition, RA group showed enhanced activities in left precuneus, left superior frontal gyrus and right cingulate gyrus under the rotation condition (Fig. 3a). RA patients in active (DAS28 ≥ 2.6) showed enhanced activation the left precuneus, left middle frontal gyrus and right cingulate gyrus compared to the patients in remission (DAS28 < 2.6) (Fig. 4, Table 4).

**Table 3:** Brain activations for the main effect of the group and task, and the interaction effect of group × task

Condition	Brain region	R/L	BA	Cluster size (voxels)	Talairach coordinate (mm)			*F*-value
					X	Y	Z	
Main effect of group								
	Precuneus	L	7	491	−25	−49	49	14.39
		R	7	120	6	−60	49	9.52
	Superior frontal gyrus	R	9	251	−23	24	49	15.07
	Cingulate gyrus	L	24	71	−10	3	49	4.57
Main effect of task								
	Precuneus	L	7	2081	−24	−71	34	10.67
		R	7	763	27	−67	34	7.62
	Medial frontal gyrus	R	10	112	8	52	27	18.29
		L	10	96	−3	57	21	22.69
	Anterior cingulate	R	32	46	9	14	34	9.15
	Posterior cingulate	L	23	67	−6	−52	34	9.04
	Precentral gyrus	R	4	134	44	3	34	7.62
Interaction effect of group × task								
	Inferior frontal gyrus	R	45	50	47	41	−4	16.77
	Precuneus	L/R	7	41	−5	−50	48	9.15
	Middle frontal gyrus	R	8	38	47	13	38	10.67

*R* right, *L* left, *X, Y, Z* maximum intensity points of activation in TT coordinates (mm), *BA* Brodmann area

*p* < 0.05 (FDR-corrected); cluster size > 20 voxels

**Table 4:** Brain activations for the simple effect of the group and task and the comparison between active RA patients vs. patients in remission

Condition	Brain region	R/L	BA	Cluster size (voxels)	Talairach coordinate (mm)			*t*-value
					X	Y	Z	
RA group: rotation vs comparison								
	Precuneus	L	7	288	−19	−75	45	3.76
		R	7	149	26	−61	52	2.31
	Inferior occipital gyrus	L	18	175	−26	−89	−11	3.13
	Middle frontal gyrus	L	6	76	−23	−5	49	4.54
		R	6	69	30	−1	52	3.42
		L	6	34	−33	52	−4	2.82
HC group: rotation vs comparison								
	Middle occipital gyrus	L	17/18	178	−41	−60	−7	3.92
		R	17/18	165	33	−83	1	2.92
	Middle frontal gyrus	R	11	42	41	48	−6	4.14
	Precuneus	R	7	80	38	−66	36	2.52
		L	7	67	−24	−71	36	2.73
	Precentral gyrus	R	9	75	38	5	36	3.45
Rotation task: RA group vs HC group								
	Precuneus	L	7	164	−30	−53	52	3.24
	Superior frontal gyrus	L	8	90	−16	38	41	2.64
	Cingulate gyrus	R	24	21	2	−5	28	2.65
RA group: patients in active vs patients in remission								
	Precuneus	L	7	124	−5.2	−54	48.5	2.47
	Middle frontal gyrus	L	11	86	−33.2	44	20	2.51
	Cingulate gyrus	R	24	58	4	−48	26	2.41

*R* right, *L* left, *X, Y, Z* maximum intensity points of activation in TT coordinates (mm), *BA* Brodmann area

*p* < 0.05 (FDR-corrected); cluster size > 20 voxels

### Correlation analysis between ROIs and disease activity of RA patients

**Fig. 5:**
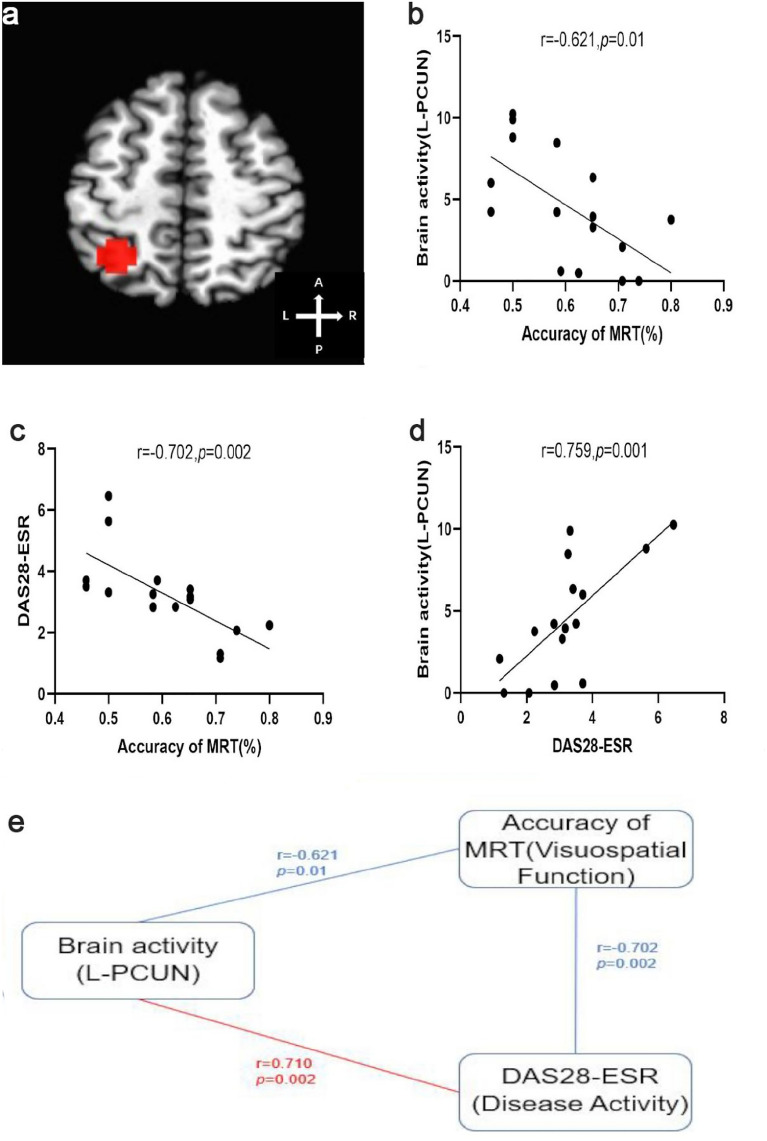
Correlation analysis involving activation of the left precuneus, MRT accuracy and DAS28-ESR in RA patients. **a** The 10 mm central diameter region of the left precuneus (L-PCUN) was selected as the ROI; **b** L-PCUN activity was negatively correlated with accuracy of the MRT; **c** DAS28-ESR was negatively correlated with accuracy of the MRT; **d** L-PCUN activity was positively correlated with DAS28-ESR; **e** the relationship between brain activity of the L-PCUN, accuracy of MRT and DAS28-ESR score

We chose the left precuneus (L-PCUN), left superior frontal gyrus and right cingulate gyrus as regions of interest (ROIs). L-PCUN activation during the rotation task was negatively correlated with MRT accuracy (*r* = −0.621, *p* = 0.01) and positively correlated with the DAS28-CRP score (*r* = 0.759, *p* = 0.001) and DAS28-ESR score (*r* = 0.710, *p* = 0.002). Accuracy of MRT was negatively correlated with the DAS28-CRP score (*r* = −0.584, *p* = 0.018) and DAS28-ESR score (*r* = −0.702, *p* = 0.002) (Fig. 5). While no correlations were observed between the BOLD signal of other ROIs and clinical and behavioral data. No correlations were noted between MRT accuracy and other clinical data and neuropsychological tests (all *P* > 0.05).

## Discussion

This study found that hyperactivity of the precuneus could be a critical neuroimaging marker of visuospatial impairment in RA patients. In addition, the enhanced activity of left precuneus is highly correlated with disease activity, which can provide a new basis for early clinical diagnosis and treatment evaluation of cognitive impairment in RA patients.

The MoCA scores of RA patients were below than 26, indicating mild cognitive impairment (MCI) [15]. As an important cognitive function, visuospatial cognition includes a variety of skills involving searching and locating objects, shifting spatial attention, holding objects in visual memory, conducting mental rotation and detecting patterns [18]. At present, some investigators have regarded visuospatial function as a predictor of global cognitive impairment and advocated it as a screening factor for MCI because visuospatial function may already have been impaired and compensated before the appearance of global cognitive impairment symptoms [19]. Some studies have reported that RA patients with cognitive impairment also have impaired visuospatial function [5, 7, 20], which is consistent with our findings. MRT result also showed the accuracy of RA patients was lower than that of HCs, which suggest that RA patients have visuospatial disturbance.

Under the MRT, both groups showed bilateral activations in the occipital–parietal–frontal network, which is consistent with previous research [21], and the corresponding brain areas are associated with visuospatial ability [22]. Visual processing begins with the transmission of visual stimuli to the lateral geniculate nucleus via the optic nerve, which then radiates to the primary visual center V1 (BA17) of the occipital cortex, and then to the visual center V2 (BA18), and finally becomes divided into the “where” dorsal pathway (occipito-parietal-prefrontal lobe) and “how” ventral pathway (occipito-temporo-prefrontal lobe), the dorsal pathway was responsible for spatial working memory and the ventral pathway for object working memory [23]. Our study showed that the activated brain regions of mental rotation overlapped with dorsal pathways responsible for spatial working memory, which further confirms that MRT is an effective paradigm for studying visuospatial cognition.

Compared to HCs, RA patients showed enhanced activation in the posterior parietal cortex (precuneus, mainly in the BA7 region), superior frontal gyrus and cingulate gyrus. These activated brain regions are involved in visuospatial cognitive processing, especially the posterior parietal cortex and superior parietal lobule, which have been shown proved to be the main brain regions involved in visuospatial cognition [24, 25]. With the development of neuroimaging, more attention has been paid to the anatomy and function of the deep anterior parietal lobule and precuneus. An fMRI study showed that performing spatially complex, two-handed coordination tasks selectively activates the precuneus and anterior cingulate cortex in both hemispheres compared to one-handed tasks [26]. The precuneus was activated during tasks requiring spatial information and Stephan et al. observed that the precuneus is more responsive during motor imagination than during actual joystick and finger movements [27, 28]. Activation of the precuneus in cognitive tasks requiring mental imagery is not limited to motor imagery, but also includes examples of visual rotation, deductive reasoning, musical processing, and mental navigation. In a PET study, visuospatial matrix rotation resulted in activation of the right dorsolateral prefrontal cortex, bilateral superior and inferior parietal lobes, extending to the anterior precuneus [29]. These studies suggest that the precuneus plays an important role in a series of highly integrated functions that are no longer thought of as simple extensions of visuospatial processes supported by the lateral parietal cortex. Therefore, we selected the left precuneus(L-PCUN) as an ROI for further analysis, in an attempt to find a neuroimaging marker of visuospatial dysfunction in RA patients.

RA patients performed poorly in behavioral visuospatial tasks, but in the mental rotation task, the bilateral posterior parietal cortex and dorsolateral prefrontal cortex were not only activated, but left hemisphere dominance was shown, contradicting the right hemisphere dominance in visuospatial cognition [30]. We hypothesized that this might be the neural mechanism of visuospatial cognitive impairment in RA patients. Combined with the theory of neural efficiency [31], participants showed reduced brain activity when they performed simple tasks compared to when they performed complex tasks. This explains the experimental results in this study, that is, when visuospatial cognitive processing of RA patients is impaired, mental rotation becomes a relatively complex and difficult task, so the brain needs to consume more resources to complete this task. To further test this theory, we analyzed the correlation between L-PCUN, MRT accuracy and disease activity (DAS28) of RA patients, and showed the lower MRT accuracy, the higher the disease activity and the more enhanced activation of the L-PCUN. At the same time, patients with higher disease activity had stronger activation in the L-PCUN. This is consistent with the neural efficiency conclusion mentioned above: RA patients with visuospatial dysfunction need to consume more brain resources when performing MRT, and the corresponding brain areas consumed more oxygen and are more activated. As previously mentioned, the precuneus plays a key role in the integration of visual and spatial information and occupies a central position in many higher levels of cognition [28, 29]. Abnormal precuneus activity is thought to be a predictor of MCI [8]. In this study, the abnormal activation of the precuneus may be a neuroimaging marker of visuospatial dysfunction in RA patients.

Moreover, the left precuneus showed enhanced activation in patients with active RA, compared to those in remission. Studies have shown that disease activity is associated with cognitive impairment in RA patients, and it is considered necessary to timely adjust the treatment plan of RA patients through DAS28 monitoring [32, 33]. Our study also concluded that the activation of the precuneus higher in patients with higher disease activity, suggesting that the precuneus may be a reliable radiographic indicator of disease activity in patients with RA. Some researchers believe that the infiltration of inflammatory cells in RA patients leads to the increased load of small vessels and affects the supply of blood oxygen to the central nervous system, thus causing cognitive dysfunction [3]. We also observed that the average hemoglobin (HB) of RA patients is lower than normal. Shah et al. conducted a cognitive assessment on 793 elderly community residents, and showed that both low and high hemoglobin levels are associated with cognitive dysfunction, indicating that abnormal hemoglobin levels may cause cognitive dysfunction [34]. While we observed no correlation between HB and the BOLD signal of activated brain regions, this may be related to the relatively small sample size. Mental state (anxiety and depression) also could be factors of cognitive function. The scores of anxiety and depression in RA patients did not meet clinical diagnostic criteria, and we observed no correlation between HADs and BOLD signals of activated brain regions. Drugs used to control the disease, such as corticosteroids, methotrexate (MTX) relievers and biologics, have potential side effects. Gonzalez-Suarez et al. followed up patients with oral MTX for up to 4 years, and the patients gradually developed progressive dysplasia, ataxia, and cognitive dysfunction, which gradually improved after drug withdrawal [35].

The results of this study should also be considered in light of some limitations. First, the sample size of this study is relatively limited. By increasing the sample size, future studies could be examined the other potential confounding factors such as HB levels, high depression and anxiety scores. Furthermore, steroids and disease modifying anti-rheumatoid drugs in medications of RA are major influence factors in cognition function. Therefore, future studies should take these factors into account to reveal the effects of medication on cognitive dysfunction in patients with RA, which could provide meaningful guidance for clinical treatment. In this way, the mechanism of cognitive impairment in patients with RA will be clearer, which can also provide more precise guidance for the prevention and treatment of cognitive impairment in RA.

## Conclusion

In conclusion, the left precuneus is a neuroimaging biomarker of visuospatial dysfunction in RA patients. Enhanced activation of the left precuneus is strongly associated with disease activity of RA patients, which suggests the left precuneus may be an objective radiographic indicator for assessing disease severity in patients with RA. The brain functional MRI provides a valuable imaging evaluation index that can be used to evaluate the progression and potential pathogenesis of RA.

## Data Availability

The data that support the findings of this study are available from the corresponding author upon reasonable request.
